# Comparing reverse phase high-performance liquid chromatography and enzyme-linked immunosorbent assay techniques for the quantification of κ-casein B in bovine milk

**DOI:** 10.3168/jdsc.2025-0856

**Published:** 2025-11-13

**Authors:** Giovanni Niero, Claudio Cipolat-Gotet, Giorgia Stocco, Elena Mariani, Andrea Summer, Elena Visentin, Massimo De Marchi, Mauro Penasa

**Affiliations:** 1 Department of Agronomy, Food, Natural resources, Animals and Environment, University of Padova, 35020 Legnaro (PD), Italy; 2 Department of Veterinary Science, University of Parma, 43126 Parma, Italy

## Abstract

Among κ-CN variants in milk, κ-CN B is the most reactive to chymosin activity, enhancing the quality, profitability, and sustainability of the final cheese product. The aim of this study was to assess the agreement between reverse phase HPLC (RP-HPLC) reference method and a rapid ELISA technique for the quantification of κ-CN B in individual bovine milk samples. Chromatographic and immunoenzymatic analyses were performed on individual milk samples from 933 Brown Swiss cows, with κ-CN B expressed as (1) milligrams per milliliter of milk, (2) percentage of κ-CN B over total milk protein content, and (3) grams of κ-CN B yielded on a milking event. The agreement between κ-CN B phenotypes measured through RP-HPLC and ELISA was evaluated through r and z-scores. Results suggested a general agreement between the 2 techniques, with r ranging from 0.88 for κ-CN B expressed in milligrams per milliliter and as a percentage to 0.90 for κ-CN B expressed in grams. This is further supported by relatively low z-scores (<0.5), which suggested the absence of significant differences between the values obtained from RP-HPLC and ELISA. Observed discrepancies were likely because ELISA does not provide quantitative results for concentrations of κ-CN B >10 mg/mL, and to the limited sensitivity of the ELISA at low concentrations of κ-CN B. Overall, findings of the present study demonstrated a strong agreement between the 2 techniques.

## Highlights

• Chromatographic and immunoenzymatic measures for κ-CN B were strongly correlated.
• The immunoenzymatic technique showed limits at high and low κ-CN B concentrations.
• Improving the sensitivity of the immunoenzymatic technique would result in greater accuracy.

## Graphical Abstract

**Figure:**
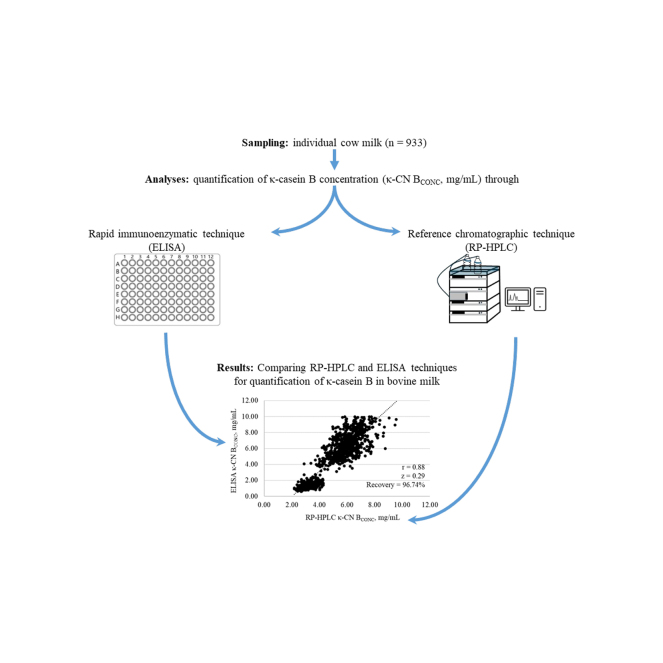
**Summary:** This study evaluated the agreement between the reference chromatographic method, reverse phase high performance liquid chromatography (RP-HPLC), and a rapid enzyme-linked immunosorbent assay (ELISA) technique for the quantification of κ-casein B (κ-CN B) in individual bovine milk samples. Results showed substantial agreement between the 2 methods, except for some discrepancies mainly due to the limitations of ELISA in quantifying very high and low concentrations of κ-CN B. Overall, ELISA can serve as a technique for routine screening or large-scale studies, thus enabling faster processing at lower costs compared with more complex analytical techniques.

The quality and efficiency of cheesemaking process are multifactorial and depend on the complex interplay of several milk traits, including gross composition, acidity, SCC, mineral profile, and protein composition (Fox et al., 2017). Among milk proteins, CN fraction plays a central role in cheesemaking, as it represents the main constituent of milk protein micelles, which in turn determine milk syneresis and curd aggregation during enzymatic coagulation. In particular, κ-CN is primarily and directly involved in the cheesemaking process because it is the specific target compound of chymosin-based rennet (Niero et al., 2024). κ-Casein exists in different genetic variants, including κ-CN A, B, C, D, and E (Sanchez et al., 2020; Guggisberg et al., 2024). Among these, κ-CN B is the most reactive toward chymosin activity, thus leading to greater cheesemaking efficiency and improved cheese quality (Schaar et al., 1985), and it seems to be associated with a greater degree of glycosylation compared with other κ-CN variants (Coolbear et al., 1996). This is reflected by an increased stabilization toward other CN fractions, greater micelle size, and prompter reactivity to chymosin (Doi et al., 1979; Bonfatti et al., 2014).

Given the important outcomes at technological level, the frequency of κ-CN alleles has been extensively investigated at the individual dairy cow level, particularly in relation to the influence of different cattle breeds (Poulsen et al., 2013; Ketto et al., 2017). Some studies suggest that genetic selection for improved milk yield and quality may have contributed to an increased frequency of the κ-casein B allele in certain cattle populations, although this relationship is not yet fully established (Kamiński et al., 2023). The study of κ-CN variants is even more relevant for countries with cheesemaking traditions (i.e., countries where milk is mainly destined for cheese production; Niero et al., 2020).

Based on this background, there is interest in developing and implementing analytical methods that are able to qualitatively discriminate κ-CN B from other κ-CN variants, and to quantitatively determine κ-CN B. Reverse phase HPLC (**RP-HPLC**) has been widely adopted for profiling detailed milk protein composition (Bobe et al., 1998; Vigolo et al., 2022) and is now considered a reference technique for both qualitative and quantitative determination of milk protein genetic variants, including κ-CN (Bonfatti et al., 2008). Although RP-HPLC offers high sensitivity and accuracy, it is also associated with considerable drawbacks in terms of labor intensity, analysis time, and operational costs.

The ELISA has been proposed as an alternative technique for fast and cheap quantification of total CN (Štumr et al., 2010) and κ-CN fraction (Ivens et al., 2016). More recently, several ELISA kits have become available for the specific quantification of κ-CN B variant (Summer et al., 2010). Nevertheless, the agreement between RP-HPLC and ELISA techniques for the quantification of κ-CN B has not been systematically evaluated. Therefore, the extent to which ELISA reflects the actual concentration of κ-CN B remains uncertain. The present study aimed to gauge the agreement between the RP-HPLC reference method and the ELISA rapid technique for the quantification of κ-CN B in individual bovine milk samples.

The study was conducted as part of the GENEtoCHEESE project (project number: D94I19000840001), which investigated the genetic and phenotypic factors influencing milk quality and cheesemaking efficiency in dairy cows. A total of 1,080 Brown Swiss cows were sampled across 54 dairy herds in Northern Italy. Each herd was visited once during the evening milking, and 20 cows per herd were sampled at each visit. Immediately after collection, individual milk samples were aliquoted for gross composition analyses and determination of κ-CN B via RP-HPLC and ELISA.

One milk aliquot was transported at 4°C to the laboratory of the Breeders Association of Veneto Region (Vicenza, Italy). Within 24 h, milk samples were warmed, gently mixed by inversion to promote solid homogenization, and analyzed for gross composition, including fat, protein, CN, and lactose content, using a MilkoScan 7DC (Foss, Hiller⊘d, Denmark) according to ISO 21543:2020 and International Committee for Animal Recording guidelines (ISO, 2020; ICAR, 2025).

The second milk aliquot was transported at 4°C to the University of Padova Department of Agronomy, Food, Natural Resources and Environment (Legnaro, Italy), where samples underwent the preparation protocol proposed by Bobe et al. (1998) before being analyzed through RP-HPLC for the quantification of κ-CN B. Milk samples were warmed at room temperature, repeatedly inverted to promote sample homogenization, and prepared for chromatographic injection according to the following procedure: (1) 500 µL of milk were mixed with an aqueous solution of guanidine (**Gdn**) HCl (6 *M* GdnHCl, 0.1 *M* bisTris buffer, 5.37 m*M* sodium citrate, and 19.5 m*M* dithiothreitol) in a 1:1 (vol/vol) ratio; (2) the mix was vortexed for 10 s and incubated at room temperature for 1 h to promote protein solubilization; (3) the sample was centrifuged at 13,000 × *g* for 10 min at room temperature to promote fat separation; and (4) the soluble fraction was collected and diluted (1:3, vol/vol) with a solution containing 4.5 *M* GdnHCl in water, acetonitrile, and trifluoroacetic acid (**TFA**; 100:900:1, vol/vol). The RP-HPLC conditions for the separation, identification, and quantification of κ-CN variants were adapted from Bonfatti et al. (2008). The RP-HPLC system consisted of an Agilent 1260 Infinity II LC (Agilent Technologies, Santa Clara, CA) equipped with a quaternary pump, a diode array detector, and a column thermostat. A reverse phase analytical column C8 (Zorbax 300SB-C8 RP, Agilent Technologies) with a silica-based packing (3.5 mm, 300 Å, 150 × 4.6 mm i.d.) preceded by a Security Guard Cartridge System pre-column (300SB-C8 Guard Cartridges 4.6 × 12.5 mm, Agilent Technologies) was used for separation. Gradient elution was performed using a mixture of 2 solvents: solvent A (0.1% TFA in water) and solvent B (0.1% TFA in acetonitrile). The flow rate was set at 0.5 mL/min, column temperature was kept at 45°C, and detection was made at a wavelength of 214 nm. Sample vials were kept refrigerated (4°C) and the injection volume was 5 μL. The total analysis time per sample was 45 min. Data acquisition and analysis were performed using Agilent OpenLab 2 CDS software (Agilent Technologies, Santa Clara, CA). Qualitative analyses for the identification of κ-CN variants were carried out using internal and external standards (Merck, Darmstadt, Germany), allowing us to classify dairy cows as homozygous for the κ-CN A allele (genotype AA, n = 45), heterozygous for the κ-CN A and B alleles (genotype AB, n = 304), or homozygous for the κ-CN B allele (genotype BB, n = 731). Quantitative analyses for assessing κ-CN B concentrations were performed using a 5-point calibration curve (R^2^ > 0.99).

The third milk aliquot was transported at 4°C to the MilCA laboratory of the Department of Veterinary Science of the University of Parma (Parma, Italy) for analysis by ELISA technique with a commercial kit (Test KAPPA 2.0, ProGnosis Biotech, Larissa, Greece). The experimental procedure was carried out as follows: milk samples were first thoroughly mixed and diluted at a ratio of 1:1,000 using the 1× assay buffer, prepared by diluting the 20× stock solution with deionized water to a final volume of 1,000 mL. Before dilution, to avoid sampling errors, any visible residue on the tips was removed by wiping with absorbent paper. The 96-well microplate was precoated with κ-CN B antigen by the manufacturer. The total volume of the well was 350 µL. For the assay, all reagents and samples were brought to room temperature. Next, 50 µL of each standard (concentrations: 0, 0.25, 1, 2.5, 5, and 10 µg/mL of κ-CN B) or prepared milk sample were pipetted into the designated wells, followed by the addition of 50 µL of the κ-CN B horseradish peroxidase-conjugated detection solution and 50 µL of the primary antibody (i.e., anti κ-CN B). After thorough mixing by pipetting 5 times, the plate was sealed with an adhesive film and incubated for 30 min at room temperature. The plate was then subjected to 4 wash cycles using 300 µL of 1× wash buffer per well, ensuring complete liquid removal between each wash. After washing, 100 µL of 3,3′,5,5′-tetramethylbenzidine chromogenic substrate was added to each well and the plate was covered and incubated for 15 min at room temperature in dark condition. The reaction was then terminated by adding 100 µL of stop solution (supplied with the kit; 15% phosphoric acid) to each well, with gentle manual mixing. Absorbance was measured at 450 nm using a plate reader (BioTek 800 TS Absorbance Reader, Agilent Technologies), with the signal being inversely proportional to the κ-CN concentration.

The κ-CN B concentrations were determined by fitting a 4-parameter logistic standard curve to the absorbance values obtained from the calibration standards. This statistical approach was listed in the assay protocol of the manufacturer. The final concentrations of κ-CN B measured through RP-HPLC and ELISA were expressed as mg/mL (**κ-CN B_CONC_**).

The following individual records were deleted from the dataset and not used for statistical analyses: (1) records associated with dairy cows identified as homozygous for κ-CN A allele (n = 45), as they were not useful for the aim of the present study; (2) records exceeding 3 SD from the mean of κ-CN B concentration measured through RP-HPLC (n = 24); (3) records of κ-CN B concentration obtained through ELISA exceeding the upper limit of quantification of 10 mg/mL, per the manufacturer's instructions (n = 70); and (4) records exceeding 3 SD from the mean of κ-CN B concentration measured through ELISA (n = 8). The final dataset consisted of 933 records comprising 273 AB and 660 BB dairy cows.

The percentage of κ-CN B over the total milk protein content (**κ-CN B_PERC_**) was calculated as

$$\kappa -\mathrm{CN}\;B_{\mathrm{PERC}},\;\%=\frac{\kappa -\mathrm{CN}\;B_{\mathrm{CONC}}\;\left(\mathrm{mg}/\mathrm{mL}\right)}{\mathrm{Total}\;\mathrm{protein}\;\left(\mathrm{mg}/\mathrm{mL}\right)}\times 100.$$

The yield of κ-CN B (**κ-CN B_YIELD_**) was calculated as

$$\begin{array}{c} \kappa -\mathrm{CN}\;B_{YIELD}\left(g\right)=\\ \frac{\kappa -\mathrm{CN}\;B_{CONC}\;\left(\mathrm{mg}/\mathrm{mL}\right)\times \mathrm{milk}\;\mathrm{yield}\left(\mathrm{mL}/\mathrm{milking}\right)}{1,000}. \end{array}$$

Descriptive statistics and r were obtained using the MEAN and CORR procedures of SAS v. 9.4 (SAS Institute Inc., Cary, NC), respectively. To evaluate the similarity between κ-CN B phenotypes measured through RP-HPLC and ELISA, the z-score (**z**) was calculated as

$$z=\frac{m-VAL_{REF}}{SD},$$

where *m* is the mean calculated for κ-CN B phenotypes measured through ELISA; VAL_REF_ and SD are the median and the SD of κ-CN B phenotypes measured through RP-HPLC, respectively. Results from 2 experimental conditions are considered equal when |z| ≤2, similar when 2 < |z| ≤ 3, and different when |z| >3 (Thompson et al., 2006).

In the original dataset comprising all the dairy cows sampled in the present study (n = 1,080), 67.7% were homozygous for the allele of κ-CN B (n = 731), 4.2% were homozygous for the allele of κ-CN A (n = 45), and 28.1% were heterozygous (n = 304). Based on these distributions, the calculated allele frequencies were 18.2% and 81.8% for κ-CN A and κ-CN B, respectively. This marked allelic imbalance may be attributed to the long-term genetic selection applied to the Italian Brown Swiss, aimed at improving milk yield and technological quality traits (ANARB, 2025). Such selective breeding is believed to have indirectly favored the B allele of κ-CN, which is associated with enhanced cheesemaking properties (Kamiński et al., 2023). In a study on the frequencies of milk protein variants in Brown Swiss bulls and cows in France, Sanchez et al. (2020) reported a slightly higher frequency of the κ-CN A allele (25.6%) and a correspondingly lower frequency of the κ-CN B allele (74.4%) than those observed in the present study. Conversely, Hallén et al. (2008) reported substantially different allele distributions, likely due to their focus on dairy cow breeds other than the Brown Swiss. In that study, κ-CN A was the most common allele, with frequencies ranging from 66% in Swedish Red cows selected for producing milk with low fat to 77% in Swedish Holstein cows. This was followed by the κ-CN B allele, with frequencies varying from 14% in Swedish Red cows selected for high fat milk to 26% in those selected for low fat milk, and by the κ-CN E allele, with frequencies extending from 6% in Swedish Holstein cows to 14% in Swedish Red cows selected for high fat milk.

Descriptive statistics for milk yield and composition are reported in Table 1. When considering heterozygous and homozygous dairy cows together (i.e., genotype AB and BB), milk yield averaged 12.73 kg/milking. Slight variations were observed within genotype, with BB dairy cows producing more milk (12.92 kg/milking) than AB dairy cows (12.26 kg/milking), further supporting the hypothesis that genetic selection for increased milk yield may have indirectly favored the presence of κ-CN B (Kamiński et al., 2023). Average contents of fat, protein, CN, and lactose across AB and BB dairy cows were 4.03%, 3.76%, 2.99%, and 4.81%, respectively, and were consistent with the values recorded separately within each genotype. Overall, milk yield and composition generally aligned with available data on Brown Swiss cows reared in lowland environments under typical intensive farming conditions (Penasa et al., 2014). Conversely, as expected, the milk yield observed here was markedly higher than that of Brown Swiss cows reared in Italian Alpine region under extensive or semi-extensive farming systems (Zanon et al., 2020). Descriptive statistics calculated in the edited dataset for κ-CN B phenotypes measured through RP-HPLC and ELISA are reported in Table 1. When considering genotypes AB and BB together, κ-CN B_CONC_ averaged 5.22 and 5.05 mg/mL, κ-CN B_PERC_ averaged 13.92% and 13.44%, and κ-CN B_YIELD_ averaged 66.55 and 64.77 g for data measured through RP-HPLC and ELISA, respectively. As expected, regardless of the analytical technique and of the measurement unit, κ-CN B was more represented in BB than AB dairy cows. Such findings agreed with those reported by Hallén et al. (2008), who investigated the effect of β-CN, κ-CN, and β-LG genotypes on the concentration of milk protein variants, reporting a κ-CN concentration of 4.99 mg/mL in BB cows and from 3.71 to 4.85 mg/mL in AB cows, respectively.

**Table 1:** Mean (SD) of κ-casein B phenotypes^1^ measured through reverse phase HPLC (RP-HPLC) and ELISA, and milk yield and composition

Trait	Genotype AB + genotype BB			Genotype AB			Genotype BB		
	Cows (n)	RP-HPLC	ELISA	Cows (n)	RP-HPLC	ELISA	Cows (n)	RP-HPLC	ELISA
κ-CN B phenotype									
κ-CN B_CONC_, mg/mL	933	5.22 (1.52)	5.05 (2.71)	273	3.27 (0.46)	1.39 (0.41)	660	6.03 (0.97)	6.57 (1.56)
κ-CN B_PERC_, %	932	13.92 (3.92)	13.44 (7.07)	273	8.73 (1.19)	3.72 (1.12)	659	16.1 (2.31)	17.5 (3.85)
κ-CN B_YIELD_, g	933	66.55 (28.12)	64.77 (40.97)	273	39.9 (13.3)	17.1 (7.42)	660	77.6 (25.1)	84.5 (31.9)
Milk yield, kg/milking	933	12.73 (3.88)		273	12.26 (3.87)		660	12.92 (3.87)	
Milk composition									
Fat, %	932	4.03 (0.79)		273	4.02 (0.77)		659	4.03 (0.81)	
Protein, %	932	3.76 (0.39)		273	3.76 (0.39)		659	3.77 (0.40)	
Fat/protein	932	1.07 (0.20)		273	1.07 (0.19)		659	1.07 (0.20)	
CN, %	932	2.99 (0.34)		273	2.99 (0.34)		659	3.00 (0.35)	
CN index, %	932	79.50 (1.42)		273	79.5 (1.40)		659	79.5 (1.43)	
Lactose, %	932	4.81 (0.22)		273	4.79 (0.22)		659	4.82 (0.22)	

1 κ-CN B_CONC_ = κ-CN B concentration; κ-CN B_PERC_ = κ-CN B percentage; κ-CN B_YIELD_ = κ-CN B yield.

Pearson correlation coefficients between κ-CN B phenotypes measured by RP-HPLC and ELISA and milk gross composition, calculated over AB and BB cows, are presented in Table 2. Milk yield had null correlations with κ-CN B_CONC_ (*P* > 0.05). Weakly positive correlations were observed between milk yield and κ-CN B_PERC_ (r = 0.12 and 0.11 for RP-HPLC and ELISA, respectively; *P* < 0.001), whereas the strongest associations were obtained with κ-CN B_YIELD_ (r = 0.62 and 0.47 for RP-HPLC and ELISA, respectively; *P* < 0.001). Fat content was weakly positively correlated with κ-CN B_CONC_, both when measured through RP-HPLC and ELISA (r = 0.14 and 0.11, respectively; *P* < 0.001). Slightly stronger correlations were calculated between protein content and κ-CN B_CONC_ measured through RP-HPLC and ELISA (r = 0.28 and 0.20, respectively; *P* < 0.001). Similar correlations were calculated between CN content and κ-CN B_CONC_ measured through RP-HPLC and ELISA (*P* < 0.001). It is likely that a certain degree of autocorrelation is present in the calculated estimates, particularly for those between κ-CN B_PERC_ and protein content, and between κ-CN B_YIELD_ and milk yield. Overall, the correlations between milk traits and κ-CN B phenotypes obtained using ELISA mirrored the pattern of associations between the same milk traits and κ-CN B phenotypes obtained through RP-HPLC. The only divergent results were observed in cases of close-to-zero and nonsignificant correlations, such as those between fat content and κ-CN B_PERC_ (r = −0.02 and 0.01 for RP-HPLC and ELISA, respectively; *P* > 0.05).

**Table 2:** Phenotypic correlations of κ-casein B phenotypes^1^ measured through reverse phase HPLC (RP-HPLC) and ELISA with milk yield and composition in AB and BB cows

Trait	κ-CN B_CONC_, mg/mL		κ-CN B_PERC_, %		κ-CN B_YIELD_, g	
	RP-HPLC	ELISA	RP-HPLC	ELISA	RP-HPLC	ELISA
Milk yield, kg/milking	0.01	0.04	0.12***	0.11***	0.62***	0.47***
Fat, %	0.14***	0.11***	−0.02	0.01	−0.06*	−0.02
Protein, %	0.28***	0.20***	−0.10***	−0.03	−0.13***	−0.06*
Fat/protein	−0.01	0.01	0.06	0.04	0.01	0.01
CN, %	0.28***	0.21***	−0.10**	−0.02	−0.13***	−0.06
CN index, %	0.19***	0.16***	−0.04	0.02	−0.04	0.00
Lactose, %	−0.01	0.01	0.06	0.06	0.21***	0.16***

1 κ-CN B_CONC_ = κ-CN B concentration; κ-CN B_PERC_ = κ-CN B percentage; κ-CN B_YIELD_ = κ-CN B yield.

* *P* < 0.05

** *P* < 0.01

*** *P* < 0.001.

Scatter plots of κ-CN B phenotypes measured in milk of AB and BB cows through RP-HPLC and ELISA revealed 2 clusters of data (Figure 1), which was expected, as it reflects the 2 distinct groups (AB and BB) considered in the study. In particular, the lower cluster comprised cows with the heterozygous AB genotype (expressing lower levels of κ-CN B), whereas the upper cluster consisted of cows with the homozygous BB genotype (expressing higher levels of κ-CN B; Table 1). Linear regressions between κ-CN B phenotypes measured through RP-HPLC and ELISA in AB and BB cows resulted in relatively strong correlation coefficients ranging from 0.88 (for κ-CN B_CONC_ and κ-CN B_PERC_) to 0.90 (for κ-CN B_YIELD_), and in relatively low z-scores ranging from 0.02 (for κ-CN B_YIELD_) to 0.38 (for κ-CN B_PERC_), highlighting a substantial agreement between the reference and the rapid technique (Thompson et al., 2006). This agreement was further supported through the similar average values of κ-CN B phenotypes measured through RP-HPLC and ELISA, namely 5.22 and 5.05 mg/mL for κ-CN B_CONC_, 13.92% and 13.44% for κ-CN B_PERC_, and 66.55 and 64.77 g for κ-CN B_YIELD_ (Table 1). The recovery of ELISA technique (i.e., the percentage ratio between κ-CN B phenotypes measured through ELISA over κ-CN B phenotypes measured through RP-HPLC), was 96.74% for κ-CN B_CONC_, 96.55% for κ-CN B_PERC_, and 97.33% for κ-CN B_YIELD_ (Figure 1). Recovery values close to 100% further highlight the agreement between the 2 studied techniques. The minor discrepancies existing between the 2 methods can be attributed to multiple contributing factors. The first is related to the upper quantification limit of the ELISA (10 mg/mL). As shown in Figure 1A, data distribution is cut off at high κ-CN B concentrations, which likely contributes to the divergence between ELISA and RP-HPLC measurements in this range. The second factor is that ELISA seemed to exhibit limited sensitivity at low κ-CN B concentrations. This is supported by Table 1, which shows systematic underestimation in this range (i.e., in AB cows), and by Figure 1, where ELISA measurements (y-axis) display lower phenotypic variability than those obtained by RP-HPLC (x-axis).

**Figure 1:**
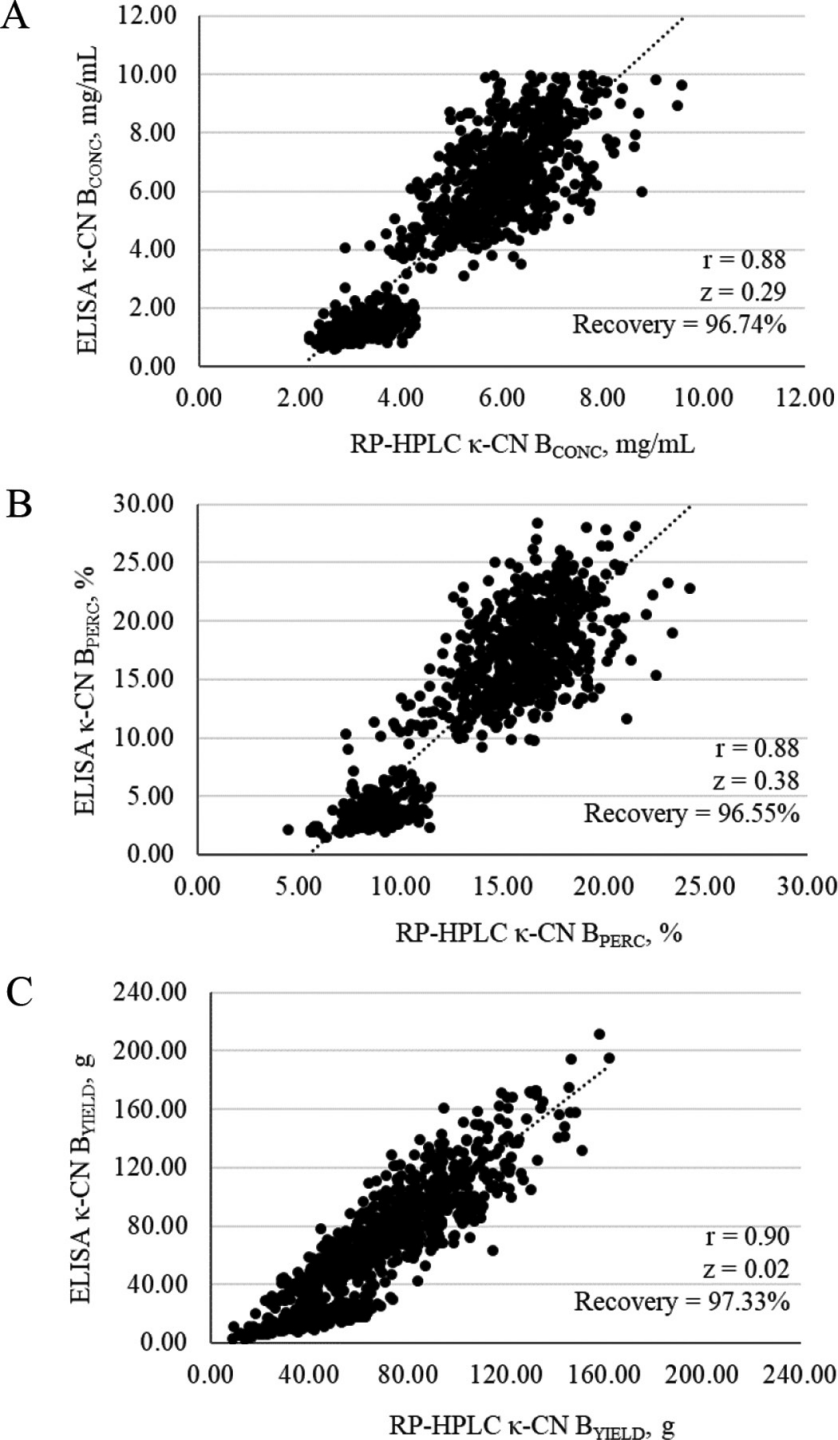
Scatter plots, r, z-scores (z), and recovery for (A) κ-CN B concentration (κ-CN B_CONC_), (B) κ-CN B percentage (κ-CN B_PERC_), and (C) κ-CN B yield (κ-CN B_YIELD_) measured through reverse phase HPLC (RP-HPLC; x-axis) and ELISA (y-axis) in AB and BB genotype cows.

In conclusion, results from this study indicate a strong agreement between the RP-HPLC reference method and the ELISA rapid assay for quantifying κ-CN B in individual bovine milk samples. Correlations coefficients ranged from 0.88 for κ-CN B_CONC_ and κ-CN B_PERC_ to 0.90 for κ-CN B_YIELD_, and z-scores were low, ranging from 0.02 to 0.38 for κ-CN B_YIELD_ and κ-CN B_PERC_, respectively. The divergences between the 2 analytical methods were likely due to the upper limit of quantification of the ELISA, which does not provide punctual quantification for κ-CN B concentrations above 10 mg/mL, and to the limited sensitivity of the ELISA at low concentration of κ-CN B. Improving performances of ELISA at high and low concentrations of κ-CN B is advisable to reach more accurate results. Enhancing the analytical performance of the ELISA test may be achieved by adjusting sample dilution. In particular, higher dilution could overcome current limitations at high κ-CN B concentrations, whereas lower dilution may improve accuracy at low concentrations. In this view, future trials should be conducted to evaluate method linearity and sensitivity in response to dilution changes. Given its consistency with the reference method, ELISA could be a useful tool for large-scale screening of dairy cows for the κ-CN B allele, especially in countries where milk is primarily destined for cheese production.
